# Pre-Pandemic Adversity Buffers the Role of Social Loneliness in Caregiver Mental Health During the COVID-19 Pandemic

**DOI:** 10.3389/ijph.2022.1604675

**Published:** 2022-08-11

**Authors:** Emma Kahle Monahan, Julie S. McCrae, Simeon Daferede

**Affiliations:** Chapin Hall at University of Chicago, Chicago, IL, United States

**Keywords:** loneliness, mental health, resilience, longitudinal study, COVID-19 pandemic, child caregiver

## Abstract

**Objectives:** This study investigates how family profiles of risk and resilience established prior to COVID-19 are associated with changes in caregiver depression and stress 1 year after the pandemic onset, and how these associations are moderated by experiences of social loneliness.

**Methods:** A sample of 243 caregivers in four risk and resilience profiles interviewed pre-COVID-19 were interviewed virtually in December 2020–February 2021 (during pandemic). Multi-level models were used to examine changes in mental health.

**Results:** All caregivers reported increases in extreme stress during the pandemic. Caregivers with less relative adversity pre-pandemic showed significantly greater depression and loneliness in the pandemic compared to caregivers with higher pre-pandemic adversity. Social loneliness was a moderator of the association between pre-pandemic adversity and mental health.

**Conclusion:** The study suggests families with more pre-pandemic adversity demonstrate coping that buffers the negative impact of social loneliness on mental health, emphasizing the strengths of these families that are assets to build upon in crisis. Families with more relative advantage pre-pandemic likely need assistance to reduce feelings of stress and depression in the face of increased social loneliness.

## Introduction

### COVID-19 Impacts on Caregiver Mental Health

While the mental health toll of the COVID-19 pandemic has been significant across all populations [1], evidence suggests it was particularly difficult for caregivers and parents. Unpaid adult caregivers experienced disproportionately worse mental health outcomes, increased substance use, and elevated suicide ideation [2]. The consequences of the pandemic have affected parents’ well-being and heightened incidents of anxiety and mental health disorders [3].

Additionally, the COVID-19 pandemic introduced global experiences of social isolation. The inability to see close friends and family and being forced to work and provide childcare within the home likely added significant additional stress to caregivers. In previous pandemics, researchers warned of the potential anxieties resulting from quarantining from a deadly infectious disease (Maunder, 2009). Indeed, studies of prior pandemics found links between social isolation and adverse health consequences, including depression (Hawley & Capitanio, 2015). Specific to COVID-19, one study across four continents found that the COVID-19 home confinement had a negative effect on the mental and emotional wellbeing of adults [4]. Another study of Italian parents confirmed that the sudden difficulty of quarantine played a crucial role in exacerbating the existing stressors that caregivers were already facing [5]. As such, the current study examines changes in caregivers’ mental health outcomes since the onset of the COVID-19 pandemic, paying particular attention to the role of social isolation in mental health changes.

### COVID-19 Impacts on Families With Varying Histories of Adversity

COVID-19 affected all families in the United States, but the disruptions of the pandemic have disproportionately impacted the well-being of families who have historically faced greater adversity, particularly families of color and low-income families. Families of color and low-income families are more likely to work in industries that experienced significant lay-offs and furloughs, and they are more likely to work in industries that require in-person work, consequently increasing risk of COVID-19 exposure and transmission ([6]; Hardy and Logan 2020).

As a result, pre-existing inequities have been exacerbated by the COVID-19 pandemic. All of these stressors can heighten feelings of anxiety, depression, and other mental health challenges. Indeed, Czeisler et al. [2] reported that the percentage of individuals considering suicide was significantly higher among racial minority groups, particularly for Hispanic and Black individuals. Another study found depression and psychosocial stress were higher among Hispanic adults [7]. Similarly, parental stress was found to be particularly high among low-income families in the wake of the COVID-19 pandemic (Creswell et al., 2021). These findings emphasize that the pandemic’s toll on low-income families and families of color cannot be understated.

While the effects of COVID-19 have and will continue to unevenly impact Black, Indigenous, and people of color (BIPOC) and low-income households, research on resilience has found that the more experience people have with prior disasters or adversities, the more they are prepared and able to navigate systems during future struggles [8]. Indeed, some studies have found high resilience in families of color in the wake of COVID-19 and other traumatic events [6, 9]. Researchers have also found that low-income families are resilient in numerous ways and able to solve problems when they arise [10]. On the other hand, one study of low-income households’ responses to the economic shocks found that low-income households were less resilient and more likely to fall back into poverty and experience declines in well-being [11]. And as previously mentioned, some studies have found families of color to demonstrate worse mental health outcomes during the COVID-19 pandemic [2]. As such, the role of prior adversity in promoting resilience and acting as a protective factor in families’ mental health outcomes in the wake of the COVID-19 pandemic is unclear.

This leads to the question of how the impact of the COVID-19 pandemic on mental health may differ based on experiences of risk and resilience prior to the onset of the pandemic, which presents an opportunity to examine the mechanisms driving heterogeneity. On one hand, the COVID-19 pandemic has exacerbated previous disparities, so families experiencing more adversity prior to the pandemic may be having an especially hard time compared to more-resourced families. Conversely, previous adversity may build assets and knowledge about navigating service systems that makes one more resilient when facing global or significant crises like the pandemic. Additionally, forced quarantine and social isolation affected all families, and likely had unique impacts on caregivers’ mental health. Thus, the current study investigates how the mental health of caregivers have changed over the course of pandemic, and how this differs by previous risk/resilience characteristics and experiences of social loneliness.

## Study Overview

The current study extends a previous longitudinal study, Evaluating Community Approaches to Preventing or Mitigating Toxic Stress (hereafter, MTS). The MTS study was a 3-year study of pediatric healthcare innovations that was conducted in five US communities (McCrae et al., 2021). The current study focuses on three of the five communities: Palm Beach County, Florida, and Alameda and Los Angeles County, California (McCrae, Spain, and Monahan, in press). These communities had a strong foundation of partnership between pediatric health care, early childhood, and concrete resources supports for families with young children prior to the pandemic; Florida and California also had distinctly different responses to the pandemic.

The present study uses survey interview data to investigate family experiences since the onset of COVID-19. This study design is unique in that we were able to re-administer survey measures in the present data collection (January 2021) that were also collected prior to the pandemic (February 2019–February 2020). Consequently, we can investigate how family experiences have changed over the first year of the COVID-19 pandemic. Additionally, the MTS study previously conducted a Latent Profile Analysis with the full, baseline sample (*n* = 888) using measures of family risk and resilience. In the current analysis, these profiles are central for understanding how risk and protective factors prior to the pandemic are differentially associated with family experiences during the pandemic. For more details on the Latent Profile Analysis methods and characteristics of each profile, see Byers et al. [12].

## Methods

### Family Interviews

For the present study, we focused follow-up recruitment in three of the five communities involved in the MTS study. We randomly sampled from families who met the following criteria: 1) had previously consented to future contact from our study team; 2) completed all three survey waves of MTS study data collection; and 3) received care at a clinic in one of the three communities of interest. We stratified by baseline risk and resilience profile, and randomly sampled within each profile. Additionally, we over-sampled for the higher-risk classes that comprised a smaller proportion of the sample. A comparison of descriptive characteristics in the current sample to the full MTS sample suggest this sample of families is representative of its reference population. There is a similar distribution of race and ethnicity across profiles in the current sample compared to the original sample; and the patterns within age and household size are similar between the two groups.

We interviewed 243 families. Interviews were conducted for approximately 60 min *via* phone or video call by bi-lingual, culturally-embedded field interviewers who have worked with these families since the beginning of the MTS study.

### Measures

#### Family Characteristics

Longitudinal analyses include family characteristics measured in both the MTS study and the current extension study. In the MTS study, information was collected about caregiver and target child age, household size, race, ethnicity, and years spent in the United States. We derived new caregiver age and years spent in the United States variables that were adjusted to reflect the time elapsed between baseline survey data collection in the MTS study and data collected for the current study. From the most recent data collection, we measured caregivers’ education level (high school or less, associates, college or more), income (standardized), and employment status (employed, unemployed).

As mentioned above, previous analyses in the MTS study identified four family profiles of risk and resilience [12]: 1) complex risk, low strengths; 2) household/relational risk, low strengths; 3) neighborhood risk, high strengths; and 4) low risk, high strengths. These family profiles are used as key predictors of outcomes in longitudinal models. Measures of risk used in the Latent Profile Analysis include neighborhood danger and disorder, housing instability, environmental adversity, and the impact of stress. Measures of resilience included mastery, social connection, agency, and a standardized resilience assessment. The complex risk, low strengths profile consisted of only 17 families in our current sample, and this group size was too small for longitudinal, multi-level models. As such, we combined the complex risk, low strengths profile with the household and relational risk, low strengths profile for longitudinal modeling only. We justified this combination because these two profiles are similar in that they both report lower strengths compared to the other two classes. Similarly, an examination of mean differences across the profiles found the complex risk, low strengths profile and the household/relational risk, low strengths profile to be similar on many demographic characteristics and outcomes (see Table 1).

**TABLE 1:** Comparing means of key variables by risk and resilience profile (California and Florida, United States. 2019–2021).

	Mean (standard deviation)					Independent sample t-test					
	All (*n* = 243)	CR-LS (*n* = 17)	HR-LS (*n* = 59)	NR-HS (*n* = 43)	LR-HS (*n* = 124)	CR- LS v HR- LS	CR-LS v NR-HS	CR-LS v LR-HS	HR-LS v NR-HS	HR-LS v LR-HS	NR-HS v LR-HS
Family Characteristics											
Caregiver age (years)	29.67	29.06	27.98	29.41	30.65					**	
	(0.38)	(1.43)	(0.72)	(1.00)	(0.51)						
Target child age at baseline (months)	2.54	2.86	2.34	2.48	2.60						
	(0.10)	(0.45)	(0.19)	(0.25)	(0.14)						
Number of adults in household	2.66	2.65	3.02	2.86	2.42					**	
	(0.09)	(0.26)	(0.17)	(0.29)	(0.10)						
Number of children in household	2.46	2.41	2.83	2.47	2.29					**	
	(0.08)	(0.26)	(0.20)	(0.22)	(0.09)						
Income	20222.28	3024.88	6416.83	5999.45	34196.89					*	*
	(4314.48)	(1388.41)	(2542.78)	(2167.85)	(8187.20)						
Years in US	21.09	20.75	14.75	22.50	23.74	*			***	***	
	(0.76)	(2.20)	(1.41)	(1.69)	(1.08)						
Unemployed	0.51	0.65	0.48	0.34	0.56		*				*
	(0.03)	(0.12)	(0.07)	(0.07)	(0.04)						
High school	0.59	0.65	0.76	0.59	0.50					***	
	(0.03)	(0.12)	(0.06)	(0.07)	(0.05)						
Associates	0.23	0.29	0.17	0.36	0.20				*		*
	(0.03)	(0.11)	(0.05)	(0.07)	(0.04)						
College plus	0.17	0.06	0.03	0.05	0.29			*		***	***
	(0.02)	(0.06)	(0.02)	(0.03)	(0.04)						
White	0.09	0.00	0.02	0.00	0.18					**	**
	(0.02)	(.)	(0.02)	(.)	(0.03)						
Black	0.11	0.06	0.12	0.14	0.10						
	(0.02)	(0.06)	(0.04)	(0.05)	(0.03)						
Hispanic	0.75	0.94	0.83	0.80	0.67			*		*	
	(0.03)	(0.06)	(0.05)	(0.06)	(0.04)						
Outcomes											
Impact of stress (pre)	0.81	1.18	1.28	0.80	0.54			**	*	***	
	(0.07)	(0.30)	(0.16)	(0.17)	(0.08)						
Impact of stress (post)	0.80	1.53	1.23	0.91	0.46			***		***	**
	(0.07)	(0.34)	(0.15)	(0.16)	(0.07)						
Extreme stress (pre)	0.26	0.29	0.42	0.32	0.16					***	*
	(0.03)	(0.11)	(0.07)	(0.07)	(0.03)						
Extreme stress (post)	0.33	0.53	0.47	0.34	0.23			**		**	
	(0.03)	(0.12)	(0.07)	(0.07)	(0.04)						
Depression (pre)	0.35	0.47	0.62	0.45	0.16			*		***	**
	(0.04)	(0.19)	(0.11)	(0.11)	(0.04)						
Depression (post)	0.67	1.12	0.98	0.70	0.45			***		***	*
	(0.05)	(0.22)	(0.11)	(0.12)	(0.06)						
Moderators											
Social loneliness	3.39	3.88	4.57	3.30	2.80					***	
	(0.17)	(0.55)	(0.36)	(0.45)	(0.20)						

Note. CR-LS, complex risk, low strengths; HR-LS, household risk, low strengths; NR-HS, neighborhood risk, high strengths; LR-HS, low risk, high strengths.

Standard errors in parentheses.

*p* < 0.05, *p* < 0.01, *p* < 0.001.

#### Outcomes

Depressive feelings. Depressive feelings are measured with a two-item subscale of a larger scale measuring environmental adversity, called the Safe Environment for Every Kid (SEEK; [13]). Respondents indicated yes or no to the following questions: 1) In the past month, have you felt down, depressed, or hopeless; and 2) In the past month, have you felt very little interest or pleasure in things you used to enjoy? This resulted in a measure with a scale of 0–2. These items were adapted by Dubowitz et al. [13] from the common measure of depression, the Center for Epidemiological Studies-Depression (CES-D; [14, 15]). In our sample, this scale demonstrated a Cronbach’s alpha of 0.65 pre-COVID and 0.64 post-COVID.

Stress. A parent’s feeling of stress is measured with one item from the SEEK. Participants indicated yes or no to the question, “Do you often feel under extreme stress,” resulting in a scale of 0–1.

Impact of stress. Beyond understanding the level of stress a parent is feeling, it is also important to assess how that stress is impacting the daily functioning of caregivers. Using the Functional Impact of Toxic Stress—Parent scale (FITS-P; Moreno et al., in press), we measured the extent to which stress impacts a caregiver’s ability to manage personal behavior, think, manage their schedule, and care for their child. Participants indicated yes/no if it had been difficult to manage any of those four areas in the past month, resulting in a scale of 0–4. In our sample, this scale demonstrated a Cronbach’s alpha of 0.63 pre-COVID and 0.58 post-COVID.

Calculating percent change. All outcome measures were assessed just prior to the COVID-19 pandemic and in the current extension study. Outcome measures were operationalized using the percent change in scores from pre-to post- COVID. The range of all outcomes at both time points was increased by one to remove the possibility of zeroes in the denominator when calculating the percent change.

#### Moderator

Social loneliness. An overall sense of emotional and social loneliness is measured with a six item scale [16]. Participants indicate no, more or less, or yes to questions like, “I miss having people around me,” “I often feel rejected,” and “There are plenty of people I can rely on when I have problems.” Items are reverse-coded so a higher score indicates greater social loneliness, and the final scale has a possible range of 0–12. This information was only collected post-COVID, and in our sample, this scale demonstrated a Cronbach’s alpha of 0.57. The reliability of this measure is relatively low in this sample, but this is likely due to the unusually high endorsement of the “I miss having people around me” item (approximately 56% of caregivers said yes). Thus, while the high number of positive endorsements on this item impacts how the items on this scale correlate, it is a crucial indicator for understanding social loneliness during COVID-19 and remains included in the social loneliness moderating measure.

### Data Analysis

Descriptive analyses were conducted to examine how the covariates, outcomes, and moderating variables differ by family profile of risk and resilience.

For longitudinal analyses, multi-level modeling was used to leverage the longitudinal dataset and account for the nesting of families within the three communities. Specifically, mixed effects models were used to include both fixed effects (direct estimations of associations between predictor and outcome variables) and random effects (indirect estimates of second-level community effects).

Multi-level models were estimated to include the main effects of covariates, profiles of risk and resilience, and social loneliness as predictors of change in depressive feelings, stress, and the impact of stress. The interaction of family profiles and social loneliness was also included to identify how social loneliness during the pandemic may moderate the association between families’ risk and resilience profiles and changes in outcomes over the course of the pandemic. Using multiple demographic characteristics to predict attrition from the MTS survey, survey weights were created to adjust for potential attrition bias. These weights were applied to all multi-level models. Missing data at the item-level was rare, so we did not impute for missing data on survey items. As such, the sample size across models varies slightly (see Table 2).

**TABLE 2:** Regressing changes in outcomes from pre-to post-pandemic on loneliness and risk and resilience profile (California and Florida, United States. 2019-2021).

	% Change depression	% Change extreme stress	% Change impact of stress
Loneliness	0.11**	0.05**	0.08+
	(0.04)	(0.02)	(0.04)
High risk, low strengths	0.24	0.17+	0.12
	(0.27)	(0.09)	(0.21)
High risk, high strengths	0.27***	0.28***	0.37***
	(0.08)	(0.05)	(0.04)
Loneliness X High risk, low strengths	−0.08+	−0.04*	−0.05
	(0.04)	(0.02)	(0.05)
Loneliness X High risk, high strengths	−0.12*	−0.09***	−0.09**
	(0.06)	(0.03)	(0.03)
Caregiver age (years)	−0.01	0.00	0.00
	(0.02)	(0.01)	(0.01)
Target child age at baseline (months)	0.01	0.00	−0.03
	(0.02)	(0.01)	(0.02)
Number of adults in home	0.09***	−0.01	0.05***
	(0.02)	(0.01)	(0.01)
Number of children in home	−0.05	−0.00	0.02
	(0.05)	(0.02)	(0.03)
Income (std)	0.05	−0.02	−0.03**
	(0.06)	(0.02)	(0.01)
Years in US (std)	0.03	0.03	0.11*
	(0.03)	(0.04)	(0.05)
Associates	−0.02	−0.05	0.04
	(0.09)	(0.03)	(0.17)
College plus	−0.19	0.15***	0.17
	(0.16)	(0.04)	(0.14)
Hispanic	0.23***	0.01	−0.01
	(0.06)	(0.18)	(0.18)
Black	0.04	0.01	−0.15
	(0.17)	(0.14)	(0.16)
Other race	0.08	0.14	−0.30*
	(0.16)	(0.19)	(0.12)
Unemployed	−0.08	0.02	−0.07
	(0.06)	(0.07)	(0.09)
Constant	−0.05	−0.11	−0.26
	(0.42)	(0.15)	(0.50)
Observations	230	228	230
Groups	3	3	3
ICC of community differences	0.00	0.00	0.00

Note. Community 1 (*n* = 98), Community 2 (*n* = 110), Community 3 (*n* = 35).

Robust standard errors in parentheses.

***p < 0.001, **p < 0.01, *p < 0.05.

## Results

### Descriptive Findings

Results of mean comparisons across profiles of risk and resilience can be found in Table 1. Regarding family characteristics, families in the low risk, high strengths profile are older, have significantly more income and education, live in smaller households, and are more likely to be white. Interestingly, families with high neighborhood risk and high strengths prior to the pandemic show the lowest rate of unemployment during the pandemic. These families also report the highest rate of completing an associate’s degree. Families in the high household risk, low strengths profile have spent the fewest years in the United States, and the complex risk, low strengths profile is composed of almost entirely Hispanic families (94%).

The complex risk, low strengths and high neighborhood risk, low strengths profiles reported an increase in the impact of stress over the course of the pandemic, and the other two profiles reported a small decrease. Not surprisingly, all families reported an increase in feeling extremely stressed, but the increase was largest (83%) for families in the complex risk, low strengths profile. Similarly, families in all profiles reported fairly large increases in depressive feelings (ranging from 56% to 181%). Interestingly, families in the lower risk, high strengths profile reported the largest increase in depressive feelings. Regarding social loneliness, families in the low risk, high strengths profile reported the lowest levels of loneliness, and families in the high household risk, low strengths profile reported the highest.

### Multi-Level Model Findings

Results of multi-level models can be found in Table 2. As a reminder, due to the small group sizes, we combined the complex risk, low strengths profile with the high household risk, low strengths profile for multi-level models. This combined profile is called “high risk, low strengths” in Table 2. This profile and the high neighborhood risk, high strengths profile are compared to the low risk, high strengths profile in all models. For the multi-level, longitudinal models, outcomes are interpreted as the percent increase or decrease in an outcome associated with a one-unit change in the predictor variable.

Results show a one-unit increase in social loneliness is associated with a 11% increase in depression and a 5% increase in feelings of stress, regardless of risk and resiliency profile. Families in the high risk, low strengths profile are not significantly more likely to report increases in depression or stress compared to families in the low risk, high strengths profile. Alternatively, belonging to the high risk, high strengths profile is associated with a 27% increase in depressive feelings, 28% increase in feeling extremely stressed, and a 37% increase in the perceived impact of stress compared to the low risk, high strengths profile.

Interactions between social loneliness and family profiles of risk and resilience suggest social loneliness does moderate some of the association between experiences of risk and resilience prior to the pandemic and changes in mental health outcomes. Specifically, families reporting greater social loneliness and belonging to the higher-risk profiles show decreases in all outcomes compared to families in the low risk profile experiencing greater social loneliness. These associations are significant for changes in extreme stress for both higher-risk profiles, and the associations are significant for changes in depression and the impact of stress for the high risk, high strengths profile only. This suggests that families in higher-risk profiles and experiencing greater social loneliness show a 4 and 9% reduction in feelings of extreme stress compared to families in the low-risk profile. And families in the high risk, high strengths profile experiencing greater social loneliness show a significant reduction in depression and the impact of stress compared to families in the low-risk profile.

Associations between covariates and outcomes are largely null. Of note, having more adults in the home is associated with a 9% increase in depression and a 5% increase in the impact of stress, and having a college or greater education is associated with 15% increase in feelings of extreme stress. Hispanic families are associated with a 26% increase in depressive feelings.

To assess the association between community-level differences and changes in mental health, we calculated the intraclass correlation (ICC). The ICC tells us the amount of variation in the outcome that is explained by differences in the second-level variable. Interestingly, the ICCs in the current models are all zero, suggesting no variation in changes in mental health at the individual level is explained by differences at the community level.

## Discussion

The COVID-19 pandemic was unique in that it impacted all families, though we know families of color have been disproportionately affected [17, 18]. As an adverse event that has affected the entire country, examining changes in family outcomes over the course of the pandemic offers a unique opportunity to understand how families with different experiences of risk and resilience prior to the pandemic are faring in terms of mental health and stress as the pandemic continues.

Notably, mean differences showed that families with the lowest risk and greatest strengths prior to the pandemic reported the largest increase in depression. This suggests families who had less experience with risk prior to the pandemic may have had a particularly difficult time coping mentally with the pandemic, while classes with more risk experiences may have demonstrated more resilience. These COVID-specific findings are aligned with the paradox sometimes found in studies of mental health, namely that racial and ethnic minorities sometimes report equal or better mental health than their white peers, regardless of experiencing significantly more adversity [19, 20].

Additionally, mean differences showed that families with high household risk and low strengths prior to the pandemic reported the highest levels of social loneliness during the pandemic. Caregivers in this profile reported experiences of domestic violence and harsh parenting. Given the potential increased risk for child maltreatment and domestic violence due to pandemic-related factors like increased stress, depression, and economic instability and job loss [21, 22], the social isolation and lack of exposure for mandated reporting or help-seeking in this group of families is particularly worrisome. Additional attention and resources may need to be directed to families experiencing household risk if they are feeling particularly isolated during the pandemic or future crises.

In longitudinal, multi-level models, social loneliness was associated with increases in feelings of depression and extreme stress. This is not surprising given previous associations found between loneliness and poor mental health [23]. These findings build on the emerging evidence of the toll the COVID-19 pandemic is taking on people’s mental health and social connection [24, 25].

Associations between family profiles established pre-pandemic and changes in mental health outcomes suggest families in the highest-risk profile do not report greater increases in poor mental health outcomes compared to families in the low risk, high strengths profile. This may be further evidence for families with prior histories of adverse experiences demonstrating adaptability and resilience in the face of the COVID-19 pandemic. Families in the high neighborhood risk, higher strengths profile, however, do show significantly greater increases in depressive feelings, stress, and the impact of stress compared to families in the low risk, high strengths profile. This suggests that although these families demonstrated high resilience prior to the pandemic, their previous and likely ongoing experiences of neighborhood adversity increased their likelihood of poor mental health during the pandemic, highlighting the need for communities to continue directing resources and mental health services to high-need neighborhoods [26].

The interaction between social loneliness and family risk and resilience profiles showed interesting patterns, suggesting families in high-risk profiles and experiencing higher levels of social loneliness report reductions in stress and its impact and depressive feelings compared to families in the low risk profile experiencing high levels of social loneliness. Specifically, high risk, low strengths families experiencing greater social loneliness reported a 4% decrease in feelings of extreme stress compared to families in the low risk, high strengths profile experiencing greater social loneliness. Similarly, families in the high risk, high strengths profile reported a 9% decrease in feelings of extreme stress, a 9% decrease in the impact of stress, and a 12% decrease in depression. These findings suggest families with greater risk experience prior to the pandemic are likely demonstrating resilience and adaptability in response to increased social loneliness brought about by the pandemic; this resilience in the face of significant crisis and social isolation may be lower in families with fewer experiences of adversity prior to the pandemic. This further highlights the importance of recognizing and building upon families’ strengths, particularly in times of crises [27]. Additionally, these findings suggest that families who do not often interface with community supports or resources due to relative advantage prior to the pandemic may be particularly vulnerable to struggles with stress and depressive feelings during the COVID-19 crisis.

Regarding covariates in multi-level models, Hispanic caregivers reported increases in depressive feelings compared to white caregivers. This highlights the additional mental health challenges this community may be facing [28]. Given the disproportionate impact of COVID-19 on Hispanic communities, it is particularly important to direct culturally sensitive mental health resources to Hispanic families as the pandemic continues and in future crises. Not surprisingly, caregivers with higher incomes reported better mental health outcomes over the course of the pandemic, albeit a small difference. This aligns with emerging evidence that has found families with higher income have been more protected against the hardships brought about by the pandemic [29]. Finally, caregivers with a college or greater education are associated with an increase in feelings of extreme stress compared to caregivers with a high school education or less. This aligns with another recent study that found more mental health problems in individuals with a university education [30].

### Limitations

We were unable to include a sufficient number of families in the complex risk, lower strengths family profile due to the limited number included in our original sample. However, given the consistency in our findings that families with higher relative adversity prior to the pandemic fared better than families with lower relative adversity in mental health outcomes, we anticipate the findings may not have been substantially different if all profiles had been analyzed separately.

The measures of caregiver depression and extreme stress in this study were limited and should be considered indicators and not clinical assessments. The indicators were drawn from an established clinical tool with validity to clinical measures [13], but there is likely to be some variability between the indicators used in the study and clinical measures or depression or stress. Additionally, we are relying on self-report data as proxies for mental health diagnoses, which can result in self-report bias [31].

### Conclusion

In sum, these findings underscore that all families are struggling during the pandemic. Results suggest more attention and resources should be directed to families experiencing household risk in light of the increased isolation experienced during the pandemic; and generally, communities should continue directing more resources and mental health services to high need neighborhoods experiencing significantly worse mental health outcomes during the pandemic. Importantly, findings show that families with prior experiences of adversity may demonstrate greater resiliency in the face of this public health crisis. Indeed, in our sample, families with low adversity prior to the pandemic are reporting greater increases in mental health challenges when experiencing greater social loneliness. This suggests families with fewer experiences of risk prior to the pandemic may be having a particularly difficult time in response to the increased loneliness and stress that so many have experienced since the pandemic began. These findings also highlight the variation in caregiver mental health outcomes in response to social loneliness experienced during the pandemic. Community resources and interventions should account for this heterogeneity in family experiences prior to the pandemic to better identify needs and effectively direct relevant resources.
